# Supplementary material for: Crafting moiré superlattices in twisted complex oxide–transition metal dichalcogenide heterostructures
Source: Nat Commun. 2026 Feb 21;17:3025. doi: 10.1038/s41467-026-69773-7 (PMC13036047; doi:10.1038/s41467-026-69773-7)
Supplement: Supplementary file 1 — Supplementary Information [file 41467_2026_69773_MOESM1_ESM.pdf]

Supplementary Information

Crafting Moiré Superlattices in Twisted Complex Oxide–Transition Metal Dichalcogenide Heterostructures

Rahul[1#], Puneet Kaur[1#], Jia-Yuan Sun[2#], Jun-Ding Zheng[3#], Shih-Chieh Lin[4#], Yi-De Liou[1], Chia-Chun Wei[1], Shih-Chao Chang[1], Yu-Chen Liu[1], Ru-Long Gou[1], Ting-Hua Lu[5], Yann-Wen Lan[5], Tse-Ming Chen[1], Yi-Chun Chen[1], Yung-Chang Lin[6,7], Kazu Suenaga[7], Chun-Gang Duan[3*], Wei-Ting Hsu[4,8,9*], Chih-Wei Luo[2,8,10*], and Jan-Chi Yang[1,11*]

[1]Department of Physics, National Cheng Kung University, Tainan, 70101, Taiwan.
[2]Department of Electrophysics, National Yang Ming Chiao Tung University, Hsinchu 300093, Taiwan
[3]Key Laboratory of Polar Materials and Devices (MOE) and Department of Electronics, East China Normal University, 200241, Shanghai, China
[4]Department of Physics, National Tsing Hua University, Hsinchu 300044, Taiwan
[5]Department of Physics, National Taiwan Normal University, Taipei 11677, Taiwan
[6]National Institute of Advanced Industrial Science and Technology (AIST), Tsukuba 305-8565, Japan
[7]The Institute of Scientific and Industrial Research (SANKEN), The University of Osaka, Osaka 567-0047, Japan
[8]National Synchrotron Radiation Research Center, Hsinchu 30076, Taiwan
[9]Research Center for Applied Sciences, Academia Sinica, Taipei 11529, Taiwan
[10]Institute of Physics, National Yang Ming Chiao Tung University, Hsinchu 300093, Taiwan
[11]Center for Quantum Frontiers of Research & Technology (QFort), National Cheng Kung University, Tainan 70101, Taiwan
*cgduan@clpm.ecnu.edu.cn , wthsu@phys.nthu.edu.tw, cwluoep@nycu.edu.tw, and janchiyang@phys.ncku.edu.tw

# These authors contributed equally: Rahul, Puneet Kaur, Jia-Yuan Sun, Jun-Ding Zheng and Shih-Chieh Lin

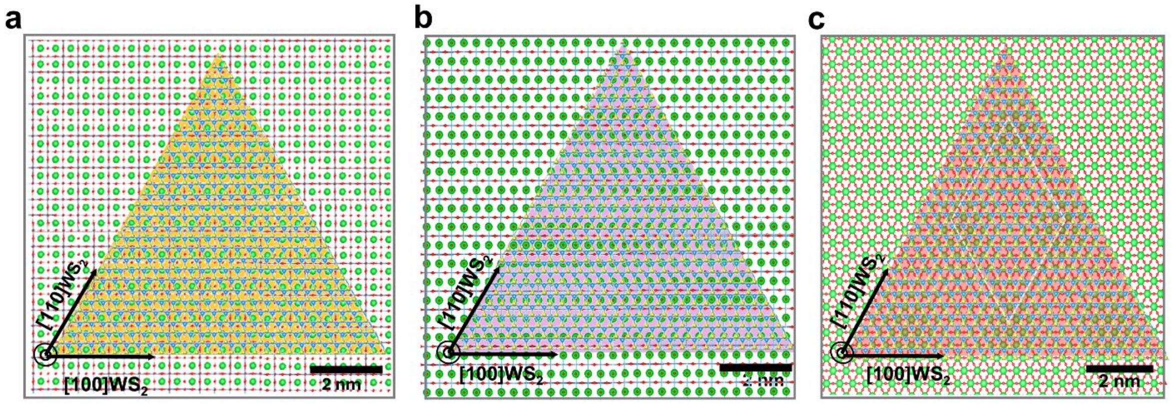

Figure S1 (a-c) Schematic representations of the stacking configurations in STO-WS_2 heterostructures for different STO orientations: (001), (110), and (111). Due to the lattice mismatch and symmetry differences, no periodic moiré patterns emerge for (001) and (110) orientations, while the (111) orientation, which shares a similar hexagonal symmetry with WS_2, facilitates the formation of distinct moiré patterns. The 0° twist angle is defined when STO $[11\bar{2}]$ is parallel to the zigzag edge of WS_2.

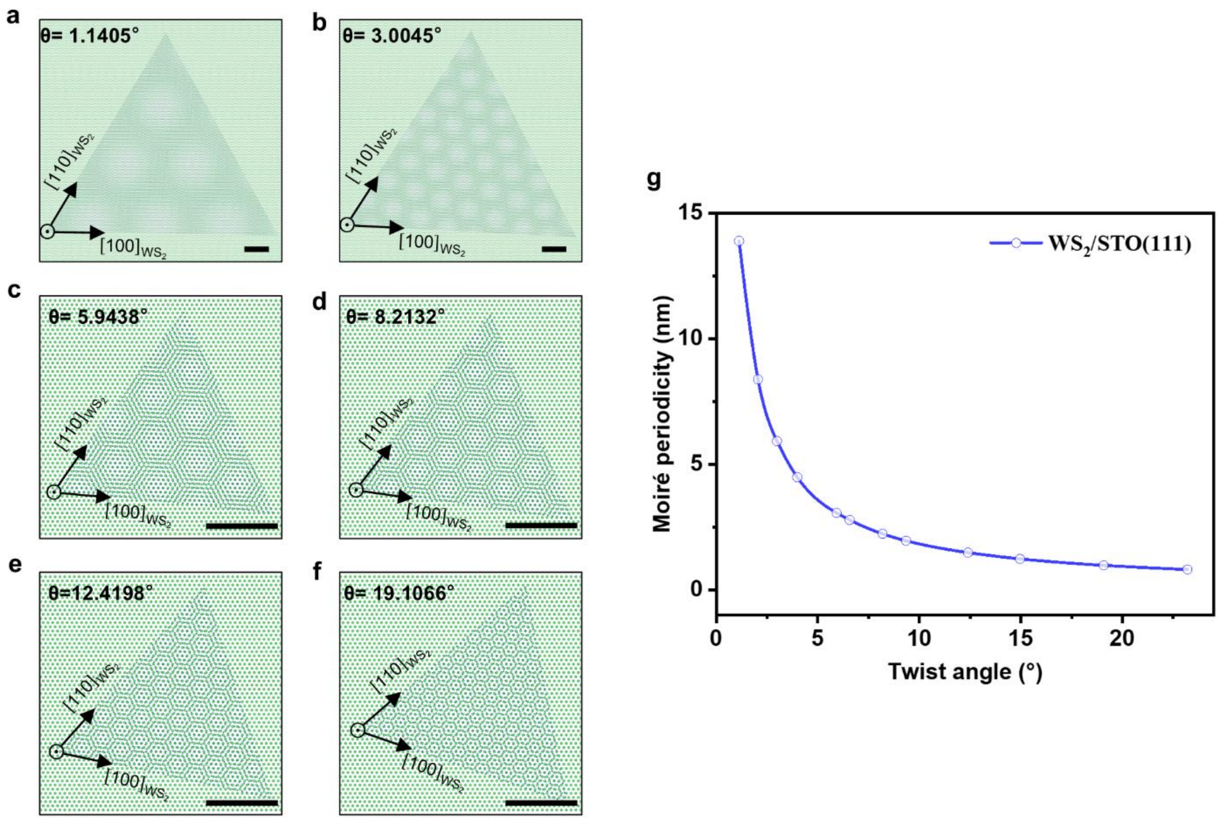

Figure S2 (a-f) Simulated moiré patterns of triangular WS_2 flakes on STO (111) as a function of relative twist angle, highlighting nearly commensurate structures that yield misfit strains below 0.5%. Sr and W atoms are denoted by green and blue spheres, respectively. The scale bar represents 5nm. (g) Simulated moiré periodicity for a twisted WS_2 flake on STO (111) as a function of twist angle.

Having identified the twist-angle dependence of moiré periodicity in WS_2/oxide heterostructures, we next performed detailed simulations of moiré patterns in a triangular WS_2 flake stacked on STO(111) across a range of twist angles (Figure S2a-f). At small twist angles, the simulated moiré superlattices exhibit large periodicities, with only a few repeating units spanning the simulated flake. As the twist angle increases, the interference pattern becomes progressively denser, forming finer hexagonal textures. While moiré patterns can, in principle, be generated at arbitrary twist angles, the emergence of well-defined, periodically repeating moiré supercells requires near-commensurate lattice matching between WS_2 and STO(111). We evaluated the misfit strain between the supercell vectors of the two lattices and selectively highlighted twist angles corresponding to supercell combinations of the WS_2 and STO(111) lattice vectors that yielded misfit strains below 0.5%. Under these conditions, the simulations reveal a robust inverse relationship between twist angle and moiré periodicity. Specifically, as the twist angle increases

from 1.14° to 19.1°, the moiré wavelength decreases systematically, following the expected inverse-angle dependence and gradually approaching saturation at higher angles. This relationship is summarized in Figure S2g, which delineates the evolution of the moiré length scale with twist angle under near-commensurate configurations. This tunable scaling behavior underscores the capability of oxide–TMD heterostructures to host controllable moiré superlattices, thereby providing a versatile platform to explore emergent electronic and quantum states in twisted systems.

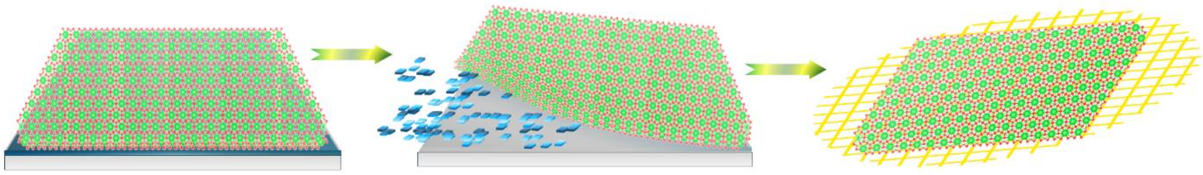

Figure S3 Schematic illustration of the transfer process of freestanding $SrTiO_3$ (FS-STO) thin films onto a TEM grid. The grey slab represents the single crystal $SrTiO_3$ (111) substrate, the dark blue layer corresponds to the sacrificial $La_{0.7}Sr_{0.3}MnO_3$ (LSMO) layer, and the green layer denotes the STO thin film. After selective etching of the sacrificial LSMO layer, the released FS-STO film is transferred onto a TEM grid, depicted as the yellow mesh, with the FS-STO resting on top. The freestanding configuration eliminates substrate contributions, enabling direct observation of intrinsic lattice periodicities and moiré superlattices when stacked with 2D materials.

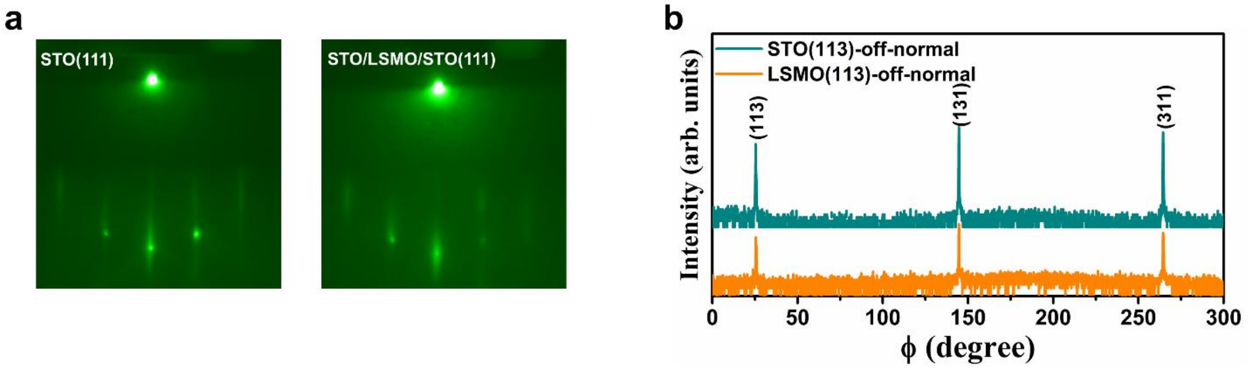

Figure S4 (a) shows the reflection high-energy electron diffraction (RHEED) pattern, where high-intensity features correspond to diffraction maxima from the crystalline STO surface and (b) X-ray diffraction (XRD) phi scan, confirming the growth of high-quality epitaxial single-crystal STO thin film.

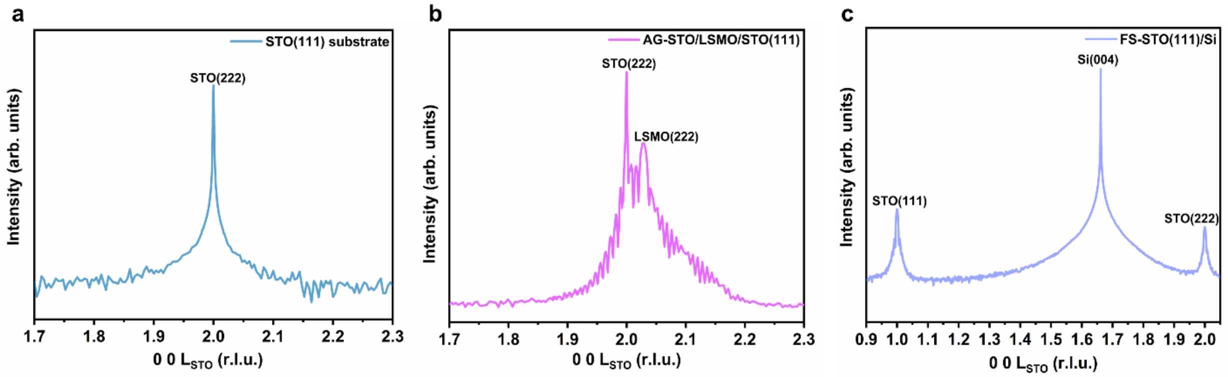

Figure S5 shows the L-scan XRD patterns of (a) bare STO (111) substrate, (b) as-grown STO/LSMO/STO (111) heterostructure, and (c) freestanding STO (111) transferred onto a Si substrate referenced with respect to STO (111). The coordinates are normalized according to the lattice parameters of STO (111), where 1 reciprocal lattice unit (r.l.u.) is defined as $2\pi/a_{STO\,(111)}$.The consistent (111) diffraction peak intensity and sharpness confirm the epitaxial growth of single-crystalline STO films and demonstrate that their structural quality and orientation are well preserved before and after the transfer process.

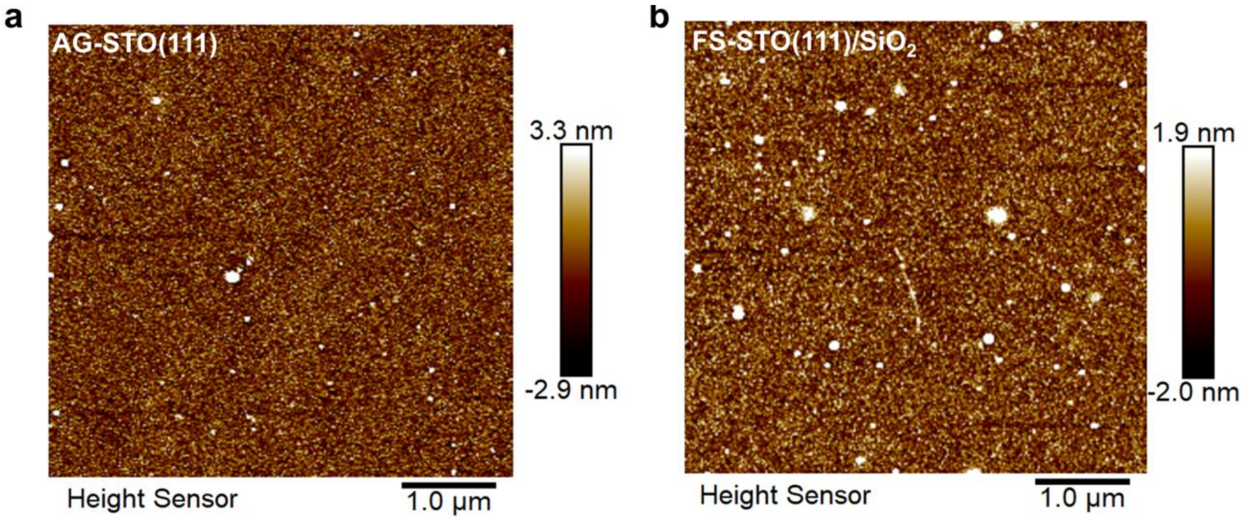

Figure S6 shows the AFM images of (a) as-grown STO/LSMO/STO (111) and (b) freestanding STO (111) transferred on /Si substrate. Before and after the transfer, the roughness of the surface remains within the nanoscale regime, indicating that the surface quality of the as-grown film remains the same after the wet chemical etching process.

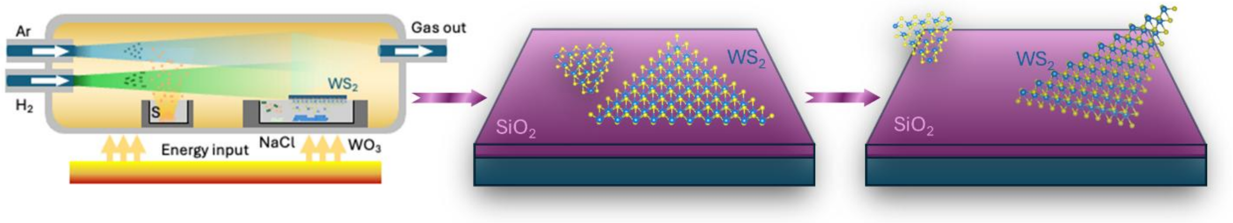

Figure S7 shows the schematic illustration of the chemical vapor deposition (CVD) growth of WS_2 and their subsequent transfer. Blue and yellow spheres represent W and S atoms in the WS_2 triangular flake, respectively, while violet color represents the underlying layer of SiO_2 on the Si substrate, which is dark grey in color.

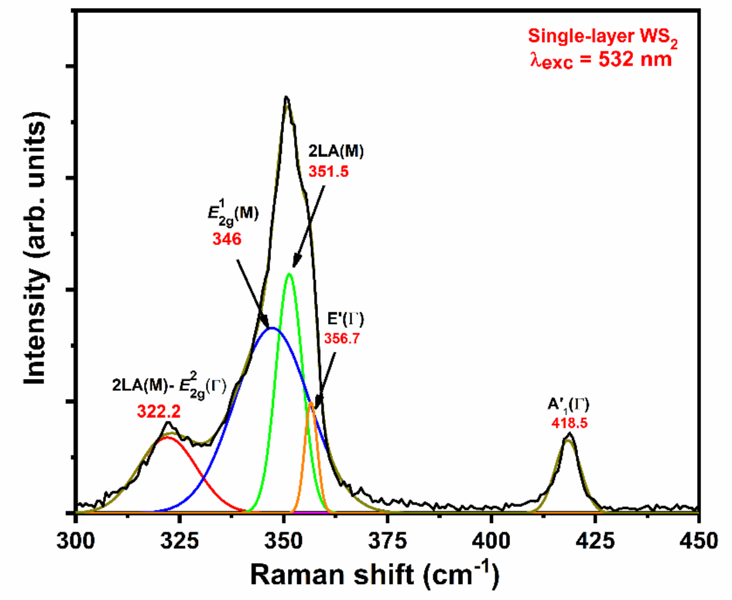

Figure S8 Room-temperature Raman spectra of monolayer WS_2. Raman spectra acquired from a monolayer WS_2 region using a 532 nm laser excitation, displaying characteristic vibrational modes of monolayer WS_2. The frequency difference between E' and A'_1 mode provides further verification of monolayer thickness. Note that the E' (in-plane) and A'_1 (out-of-plane) modes correspond to the E^1_{2g} and A_{1g} modes, respectively, in the bulk notation.

The Raman spectrum of monolayer WS_2 is dominated by the first-order modes: $E'(\Gamma)$ at 356.7 cm^{-1} and $A'_1(\Gamma)$ at 418.5 cm^{-1}. However, under 532 nm excitation, monolayer WS_2 exhibits a rich Raman spectrum with several second-order peaks that are more intense than those in the bulk. Notably, the most intense second-order feature, the 2LA(M) mode at 351.5 cm^{-1}, reaches nearly twice the intensity of the first-order A'_1 (Γ) mode. Since the 2LA(M) mode partially overlaps with

the $E'(\Gamma)$ mode near 356 cm^{-1}, their individual contributions are clearly resolved by multi-peak Lorentzian fitting (Figure S8)[1].

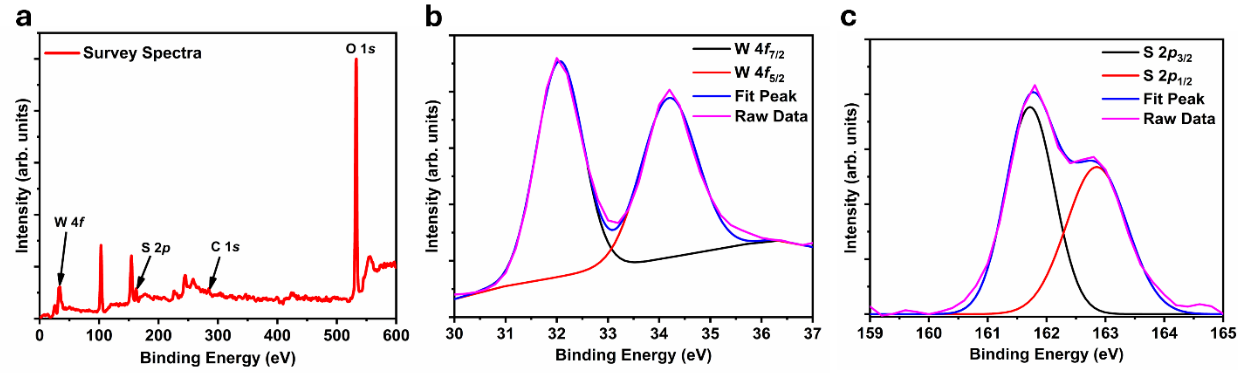

Figure S9 X-ray Photoemission Spectroscopy (XPS) analysis of the WS_2 monolayer. (a) XPS survey spectrum of the WS_2 monolayer, confirming the presence of expected elemental compositions. (b) High-resolution W 4*f* XPS spectrum showing the $4f_{7/2}$ (black) and $4f_{5/2}$ (red) doublet characteristic of W^{4+}. (c) High-resolution S 2*p* spectrum with the $2p_{3/2}$ (black) and $2p_{1/2}$ (red) components, indicating sulfur in WS_2.

X-ray photoelectron spectroscopy (XPS) is a surface-sensitive method that detects the elements present in a material and reveals their respective oxidation states. The survey spectrum shown in Figure S9a confirms the presence of C, O, W, and S. Carbon is from the atmospheric adsorption of hydrocarbons. The high-resolution plots for W and S are present in Figure S9b and Figure S9c, respectively. The deconvoluted W 4*f* spectrum shows W $4f_{7/2}$ and W $4f_{5/2}$ peaks, which correspond to the +4 oxidation state of tungsten in WS_2. Similarly, the S 2*p* spectrum exhibits S $2p_{3/2}$ and S $2p_{1/2}$ peaks, corresponding to the −2 oxidation state of sulfur. These results confirm the formation of high-quality WS_2, in agreement with the previous reports[2].

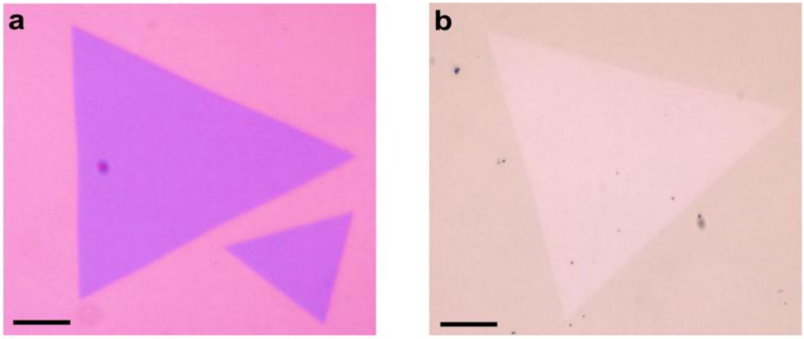

Figure S10 Optical microscopy images of WS_2 monolayers before and after transfer. (a) Optical image of triangular WS_2 monolayers synthesized via chemical vapor deposition (CVD) on a SiO_2/Si substrate, exhibiting uniform growth. (b) Optical image of a WS_2 monolayer after being transferred onto an STO (111) substrate, forming a heterostructure for subsequent characterization. The scale bar represents 10 μm.

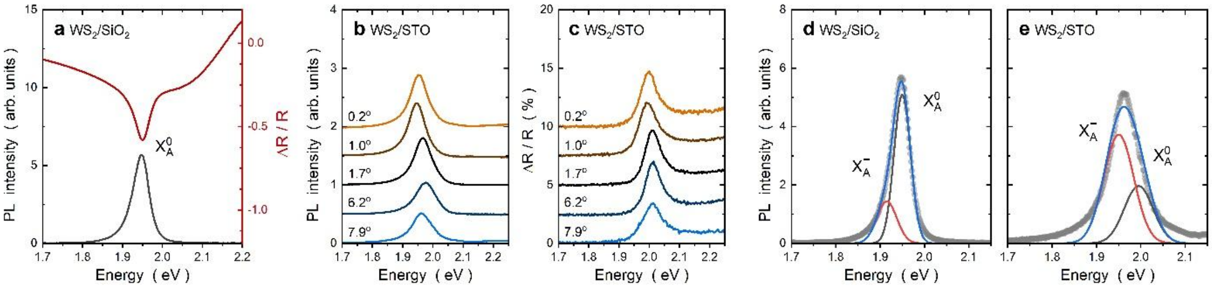

Figure S11 Room-temperature PL and DR of monolayer WS_2 on SiO_2/Si and STO. (a) PL (black curve) and DR (red curve) of as-grown WS_2/SiO_2/Si at room temperature. The main resonance at ~1.95 eV is assigned to the neutral exciton. (b, c) Room-temperature PL (b) and DR (c) of WS_2/STO. All spectra are vertically offset for clarity. The exciton energy of WS_2/STO is higher than that of WS_2/SiO_2/Si, consistent with high-κ dielectric screening. (d-e) PL spectra of as-grown WS_2/SiO_2 and WS_2/STO, fitted with WS_2 neutral-exciton (X_A^0) and trion (X_A^-) components.

We examined room-temperature PL and DR for WS_2/SiO_2 and WS_2/STO. (i) As shown in Figure S11a, for as-grown WS_2/SiO_2, the main PL peak is aligned with the DR resonance at ~1.95 eV. We therefore attribute this to the neutral exciton, a result that suggests the absence of significant carrier doping in the pristine flakes. We note that the peak energy is indeed relatively low compared to the exfoliated samples. We realize that one reason for the lower peak energy could be the residual strain generated during the CVD growth process, as previously reported[3]. (ii) For WS_2/STO (Figure S11b and Figures S11c), the PL peak is red-shifted from the DR resonance at room temperature, indicating trion emission due to interfacial charge transfer (Figure S11e). We

note that this phenomenon disappears at low temperatures, which may indicate the presence of a temperature-driven charge transfer effect. (iii) Comparing the two types of samples, the exciton energy of WS_2/STO is blue-shifted relative to the exciton energy of WS_2/SiO_2 (Figure S11d and Figures S11e), which is consistent with the recent results on the dielectric screening effect of monolayer WSe_2 in a high-κ environment[4]. Taken together, the room-temperature spectra of WS_2/STO reflect the influence of charge transfer and dielectric screening, whereas the moiré-exciton features discussed in the main text are established from low-temperature measurements, where PL and DR are energy-aligned and charge-transfer effects are minimal.

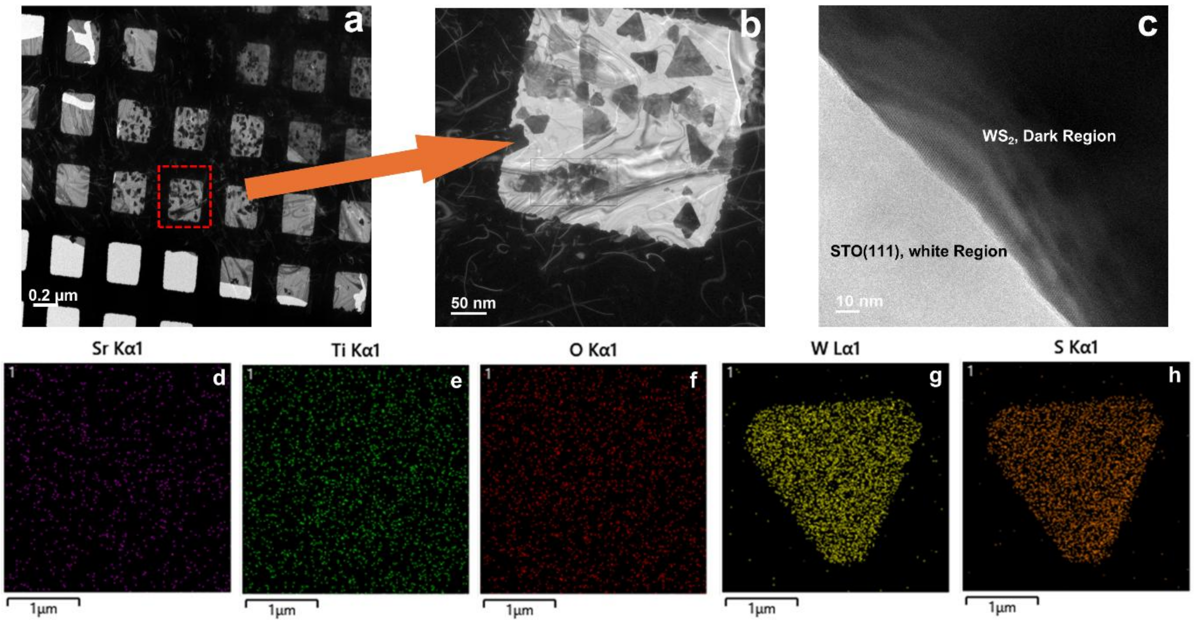

Figure S12 Transmission electron microscopy (TEM) and energy-dispersive X-ray spectroscopy (EDS) mapping of WS_2/STO (111) twisted heterostructure membranes. (a-c) TEM images showing the WS_2/STO (111) twisted heterostructure membranes on a TEM grid, illustrating the sample integrity and uniformity. (d-h) Elemental mapping using EDS, confirming the spatial distribution of constituent elements in the WS_2/STO (111) twisted heterostructure. The mapping highlights the successful transfer and integration of WS_2 monolayers onto the freestanding STO (111) membrane, demonstrating the chemical composition and uniformity of the heterostructure.

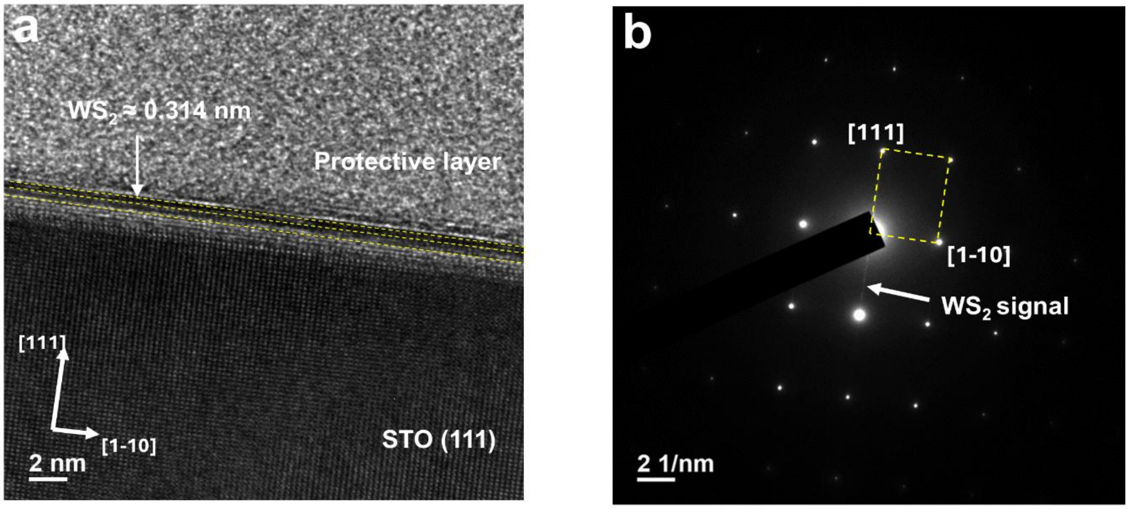

Figure S13 Cross-sectional structural characterization of WS_2/STO (111) heterostructure via high-resolution transmission electron microscopy (HRTEM) and selected area electron diffraction (SAED). (a) Cross-sectional HRTEM image of a monolayer WS_2 on an STO (111) substrate, showcasing the well-defined interface and high crystallinity of the heterostructure. The atomic arrangement at the interface highlights the structural coherence between the oxide and TMD layers. (b) Corresponding SAED pattern from the same region, confirming the crystallinity and epitaxial relationship between WS_2 and STO (111). The well-aligned diffraction spots further indicate the structural integrity and interfacial ordering in the WS_2/STO (111) twisted heterostructure.

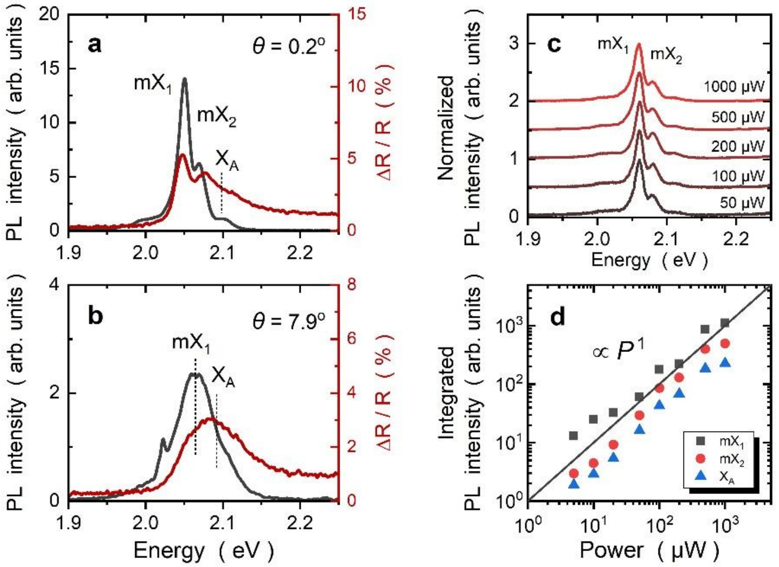

Figure S14 Moiré excitons in the $WS_2/SrTiO_3$ heterostructure. The PL (black) and DR (red) spectra measured at T = 5 K for the (a) 0.2° and (b) 7.9° twisted samples. (c) Normalized power-dependent PL spectra of the 0.2° twisted sample, showing identical exciton features at different laser powers. (d) Integrated PL intensity as a function of laser power, showing linear power dependence for mX_1 (black squares), mX_2 (red circles), and X_A (blue triangles). The black line is a reference for linear power dependence.

Figure S14a and Figure S14b compare the PL and DR spectra of the 0.2° and 7.9° samples. We observe that all exciton features, including mX_1, mX_2, and X_A, appear in both spectra. Apart from some variation in the relative intensities in the PL spectra, where lower-energy excitons are relatively stronger, the main excitonic peaks remain consistent between PL and DR measurements. Figures 3(b-c) in the main text show the evolution of DR and PL spectra as the twist angle decreases from 8° to 0°. Both mX_1 and mX_2 exhibit a redshift with decreasing angle, further demonstrating the consistency between PL and DR results. Figure S14c presents power-dependent PL measurements, showing that the relative intensities of mX_1, mX_2, and X_A remain nearly unchanged with increasing laser power. Figure S14d shows the integrated PL intensity as a function of power, showing linear power dependence for mX_1, mX_2, and X_A.

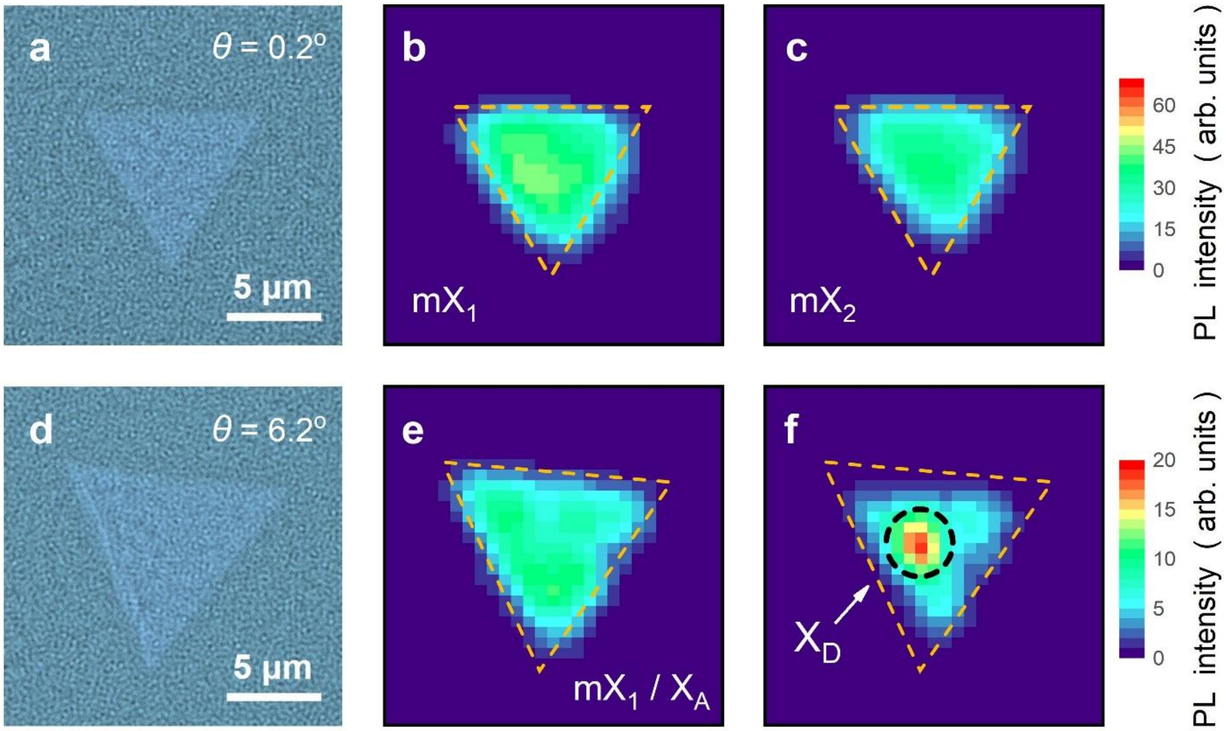

Figure S15 Uniform moiré excitons in the $WS_2/SrTiO_3$ heterostructure. Optical images of the (a) 0.2° and (d) 6.2° samples. Spatially resolved PL maps of the 0.2° (b-c) and 6.2° (e-f) samples. The PL maps reveal a uniform distribution of mX_1, mX_2, and X_A across the flakes, while the defect-bound exciton X_D exhibits clear spatial localization.

To evaluate the thermal stability and distinguish between moiré excitons and defect-bound excitons, temperature-dependent PL measurements were conducted, as shown in Figure 3(d-e) in the main text. The defect-bound exciton X_D quenches rapidly with increasing temperature, consistent with thermal escape of carriers from defect states. In contrast, the mX_1 and mX_2 peaks persist at elevated temperatures, albeit with thermally broadened linewidths. This thermal robustness supports that mX_1 and mX_2 arise from interband optical transitions, rather than from defect-related recombination. To further assess spatial uniformity and exclude extrinsic effects such as strain inhomogeneity or defects, spatially resolved PL mapping was performed. Figures S15(a-f) show optical images of the 0.2° and 6.2° samples, along with PL intensity maps of the main excitonic features, including mX_1, mX_2, and X_A. The excitonic peaks are uniformly distributed across the flakes, indicating that the underlying moiré potential is spatially homogeneous and not significantly influenced by strain inhomogeneity. In contrast, the X_D peak (highlighted in Figure S15f) exhibits strong spatial localization, a characteristic of excitons trapped at defect sites. Together, these observations confirm that mX_1 and mX_2 originate from moiré exciton states rather than from strain inhomogeneity or defects.

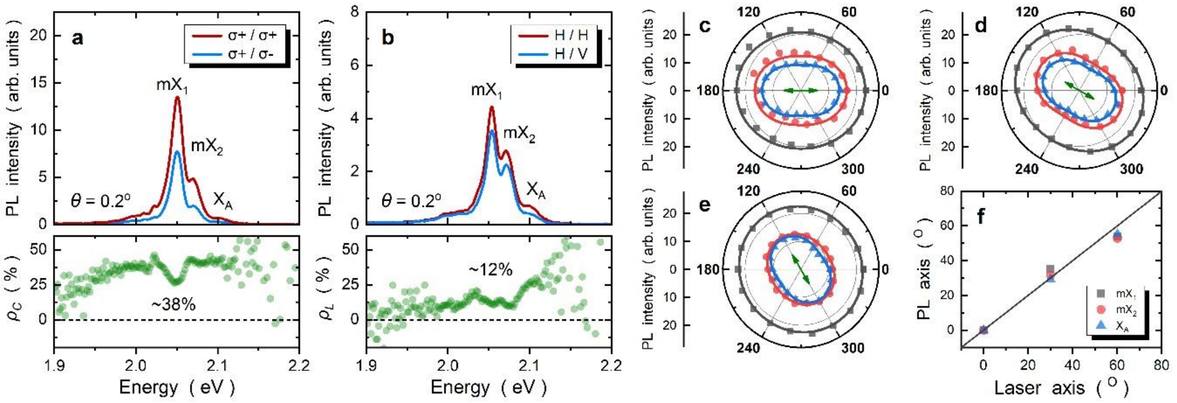

Figure S16 Polarization-resolved PL of the $WS_2/SrTiO_3$ heterostructure. (a) Circular- and (b) linear-polarization-resolved PL spectra for the 0.2° twisted sample. Upper panels: polarization-resolved PL; lower panels: degree of circular (ρ_C) and linear (ρ_L) PL polarization, defined as $((I_{(\sigma+)}-I_{(\sigma-)})/(I_{(\sigma+)}+I_{(\sigma-)}))$ and $((I_H-I_V)/(I_H+I_V))$, respectively. (c-e) Polar plots of linear-polarization-resolved PL, showing that all excitons (mX_1, mX_2, and X_A) follow the laser polarization axis. The mX_2 and X_A intensities are scaled by 2 for clarity. (f) Analysis of the PL polarization axis, showing consistent alignment with the laser polarization.

Figure S16a and Figure S16b shows the polarization-resolved PL of the 0° sample. The main excitonic features, including mX_1, mX_2, and X_A, all exhibit a certain degree of circular and linear polarization (lower panels). The circular and linear PL polarizations arise from the valley polarization and valley coherence of K-valley excitons in WS_2[5]. The finite valley coherence observed for mX_1, mX_2, and X_A demonstrates that they are K-valley neutral excitons[5]. To exclude the effects of strain inhomogeneity, we rotated the linear polarization angle of the incident laser and found that the main axis of the PL polarization rotates accordingly, as shown in Figure S16(c-f). These results demonstrate that the observed linear PL polarization indeed originates from exciton valley coherence.

We note that several important studies have demonstrated the presence of structural/atomic reconstruction in TMD moiré systems[6-12]. Critically, these microscopically reconstructed domains can significantly affect the electronic and excitonic states and may even give rise to 1D or 0D moiré structures. Although a detailed characterization of such features is beyond the scope of the present study, our linear-polarization-resolved PL measurements tentatively exclude the formation of 1D moiré structures in our sample. In a typical 1D moiré stripe phase, one would expect highly linear PL polarization with a fixed polarization axis that cannot be rotated by the incident laser. In

this case, our results are more consistent with 2D moiré excitons, which exhibit valley coherence and rotate with the incident laser polarization. These results also motivate future surface-sensitive measurements, such as PFM and STM, to directly probe the moiré pattern, which could be a valuable direction for further study.

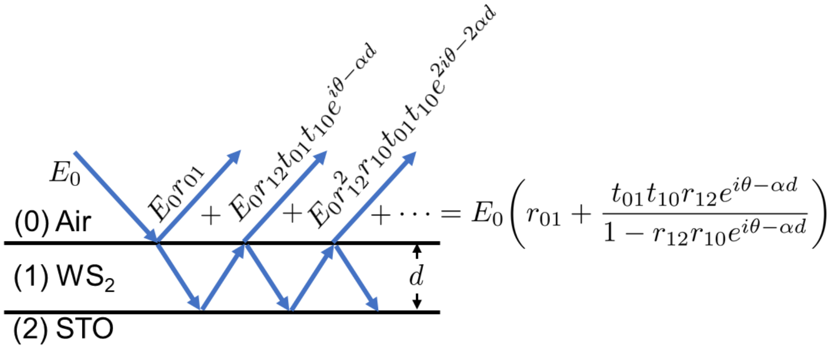

Figure S17 Reflection Contrast Model of WS_2/STO (111) twisted heterostructure. Schematic representation of the multilayer interference model used to describe the reflection contrast behavior in WS_2/STO (111) heterostructures. In differential reflectance (ΔR) measurements, the light source is normally incident on the sample surface, allowing for the extraction of excitonic features and optical transitions in the WS_2 monolayer.

As stated in the main text, the ΔR spectrum is defined as: $\frac{\Delta R}{R} \equiv \frac{(R_{WS_2} - R_{STO})}{R_{STO}}$, where R_{WS_2} and R_{STO} denote the reflected intensities from the WS_2 monolayer and the STO surface, respectively. The reflected intensity is primarily governed by multilayer interference and the Fresnel equations. As illustrated in Figure S17, normally incident white light undergoes multiple reflections and transmissions at different interfaces. We define air, the WS_2 monolayer, and the STO surface as media 0, 1, and 2, respectively. The reflection (r_{ij}) and transmission (t_{ij}) coefficients are defined as the ratios of the reflected and transmitted electric fields to the incident electric field. Assuming an incident electric field propagating from medium i to j, the reflection (transmission) coefficient is given by: $r_{ij} = \frac{(n_i - n_j)}{(n_i + n_j)} \left(t_{ij} = \frac{2n_i}{(n_i + n_j)} \right)$, where n_i represents the refractive index of medium i.

According to Figure S17, the total reflected electric field from the WS_2/STO TBL is: $E_r = E_0 r_{01} + E_0 t_{01} t_{10} r_{12} e^{i\theta - \alpha d} + E_0 t_{01} t_{10} (r_{12})^2 r_{10} e^{i2\theta - 2\alpha d} + \cdots$, where $\theta = -4\pi n_1 d/\lambda_0$ represents the phase shift through the WS_2 monolayer, with λ_0 being the incident wavelength in vacuum and $d = 0.65\, nm$. Moreover, α denotes the absorption coefficient of WS_2. Consequently, the overall

reflection coefficient of WS_2 is given by: $r_{WS_2} = E_r/E_0 = r_{01} + \frac{t_{01}t_{10}r_{12}e^{i\theta-\alpha d}}{(1-r_{12}r_{10}e^{i\theta-\alpha d})}$. Similarly, the reflection coefficient of the STO surface is simply: $r_{STO} = r_{02}$. Since we only fit the DR spectra near the A-exciton resonance, a non-dispersive refractive index $n_2 = 2.4$ was used for STO, as referenced in[13]. For WS_2, the excitonic transitions can be modeled using Lorentz oscillators, where the refractive index is derived from the complex dielectric function: $\tilde{\varepsilon}(\omega) = 1 + \sum_k \frac{A_k^2}{\omega_k^2-\omega^2-i\gamma_k\omega}$, which satisfies the Kramers-Kronig relation. Here, A_k and γ_k correspond to the oscillator strength and linewidth of the kth Lorentz oscillator[14], while ω_k represents the natural oscillation frequency, which corresponds to the exciton energy. In this work, we fit the A-exciton (X_A) and moiré excitons (mX_1 and mX_2) using three Lorentz oscillators.

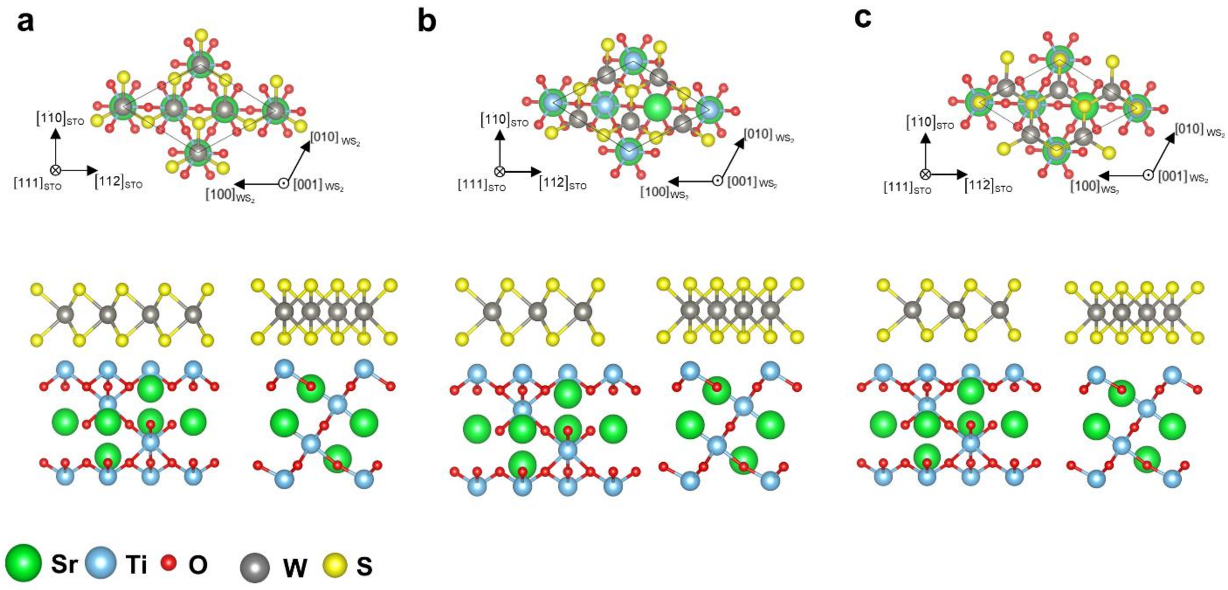

Figure S18 Atomic registries of (a) AA, (b) AB, and (c) AC stacking configurations.

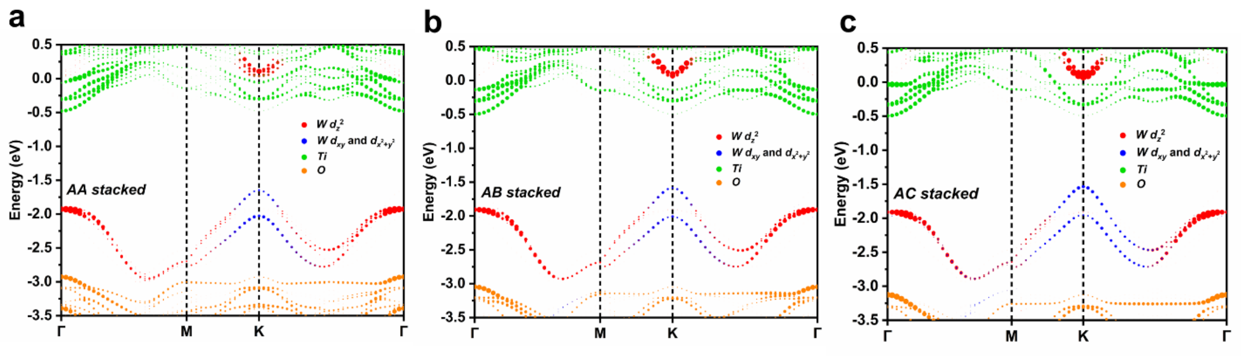

Figure S19 DFT calculation of $WS_2/SrTiO_3(111)$ with high symmetry stacking sites. Band structures of (a) AA, (b) AB, and (c) AC stacking configurations.

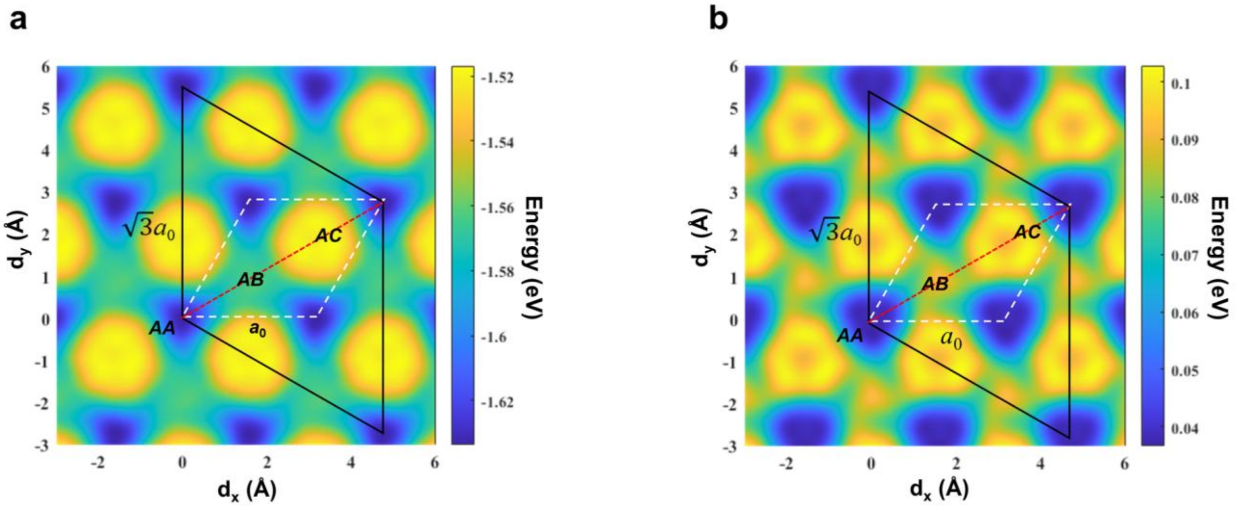

Figure S20 DFT calculation of $WS_2/SrTiO_3(111)$ with different stacking sites. DFT calculations of the (a) valence-band shift and (b) the conduction-band shift.

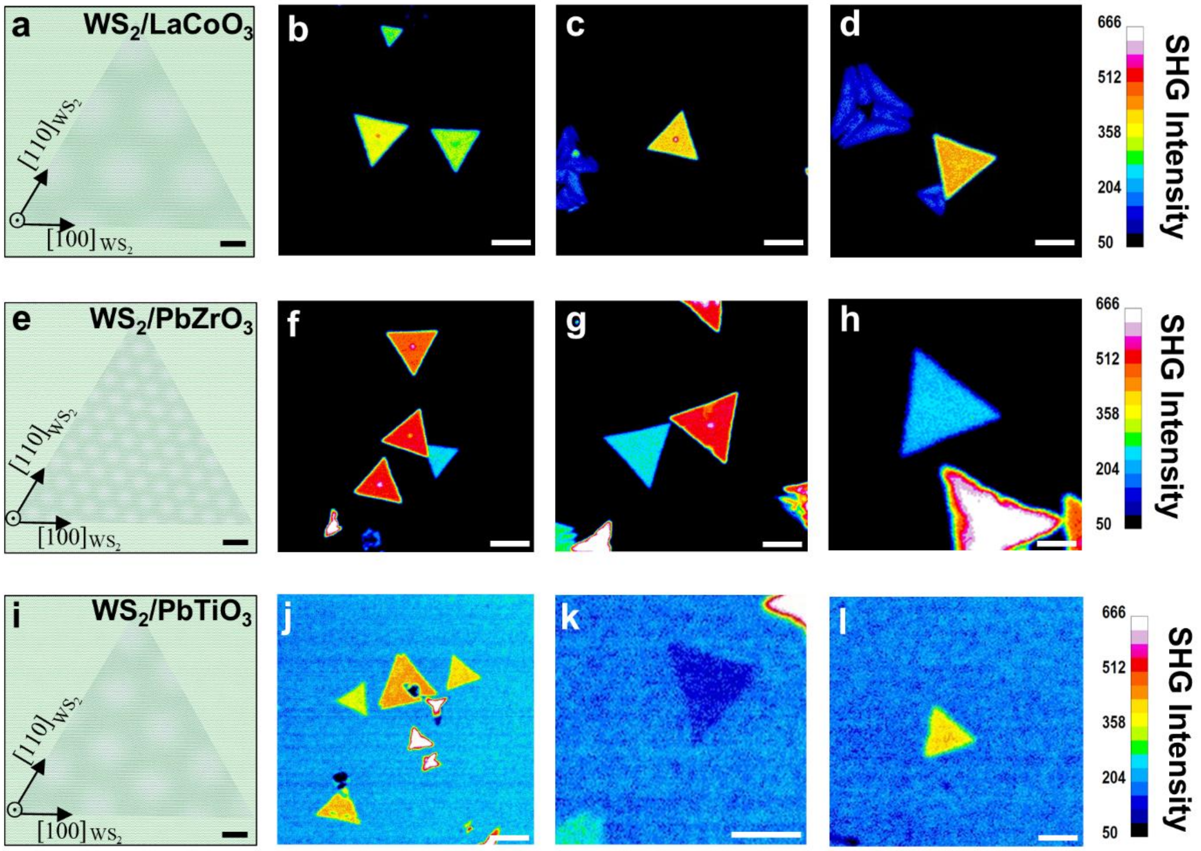

Figure S21 Schematic illustrations and SHG images of (a-d) $WS_2/LaCoO_3$, (e-h) $WS_2/PbZrO_3$, and (i-l) $WS_2/PbTiO_3$. Scale bars for moiré illustration and SHG are 5 nm and 20 μm, respectively.

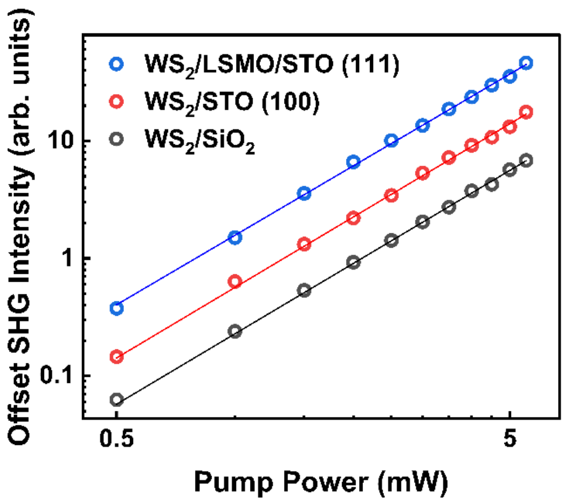

Figure S22 SHG intensity of WS_2 on LSMO (111), STO (100), and SiO_2 substrate as a function of pumping power, fitted with a quadratic function (solid lines).

SHG intensity as a function of pumping power for WS_2 on various perovskite oxide materials. The data were fitted using the function $y = ax^b$, yielding exponent values of b = 1.99 ± 0.08 for WS_2 on STO (111), 1.99 ± 0.05 on Si, and 1.96 ± 0.05 on LSMO (111). These results confirm that the SHG field scales quadratically with the incident field strength, the principles of nonlinear optical theory[15].

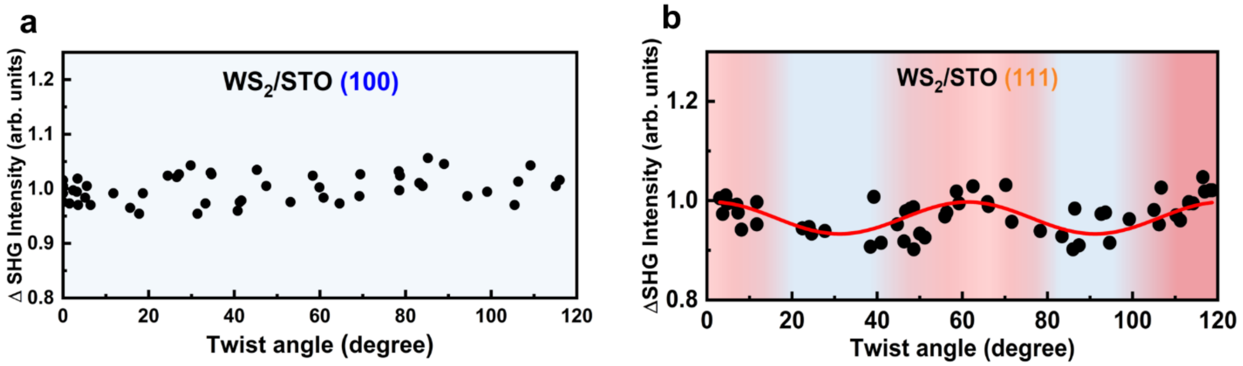

Figure S23 Twist angle-dependent normalized SHG intensity changes (ΔSHG) for WS_2 on various substrates with different orientations, including (a) WS_2/STO (100), and (b) WS_2/STO (111).

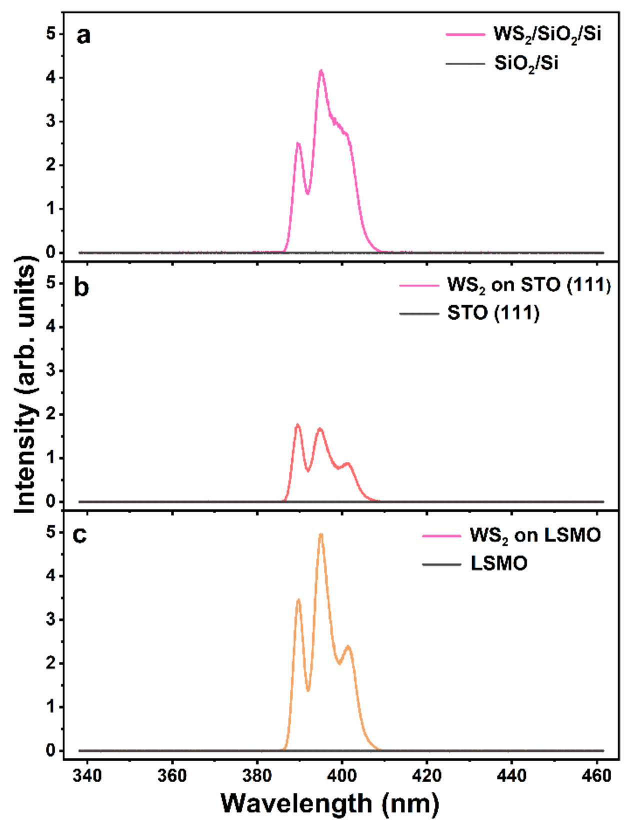

Figure S24 SHG spectra of (a) WS_2/Si, (b) WS_2/STO(111), (c) WS_2/LSMO, along with the corresponding bare substrates (Si, STO(111), and LSMO) for comparison.

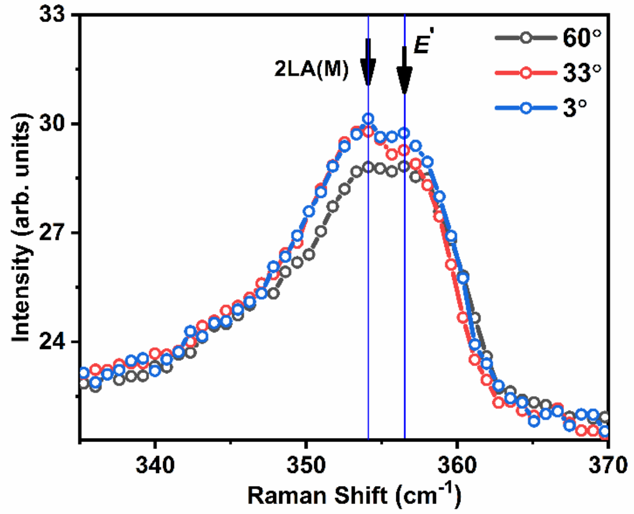

Figure S25 Raman spectra of twisted WS_2/LSMO(111) heterostructures at twist angles of 3°, 33°, and 60°. Note that the E' mode corresponds to the E_{2g}^{1} mode in the bulk notation.

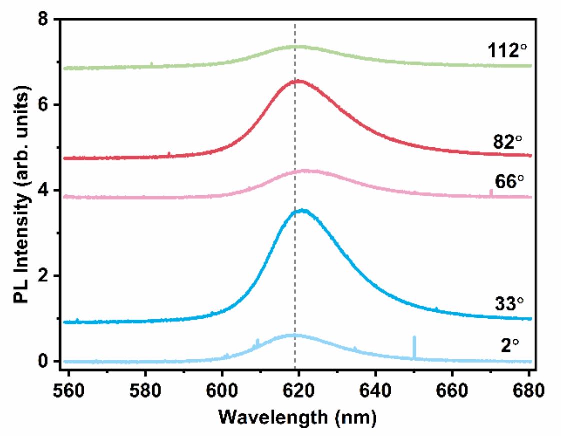

Figure S26 Twist angle-dependent photoluminescence (PL) intensity of WS_2/LSMO/STO (111) twisted heterostructure.

The twist angle-dependent photoluminescence (PL) measurement reveals significant PL quenching at smaller interlayer distances, such as at the twist angles of 0° and 60° [16,17] as shown in Figure S26. This result supports the argument that the charge transfer efficiency is modulated by the variations in interlayer spacing directly. Therefore, the observed modulation in SHG intensity could be attributed to the variation of interlayer spacing induced by the twist angles.

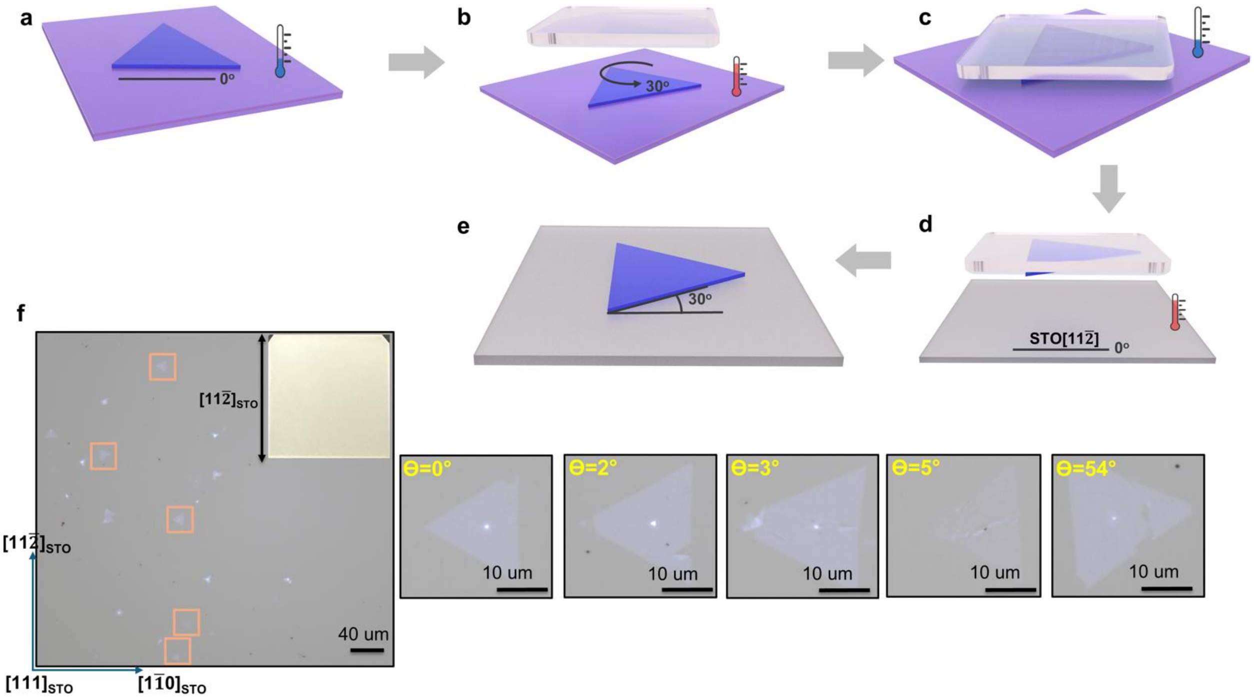

Figure S27 Schematic illustration of the PCL-based dry-transfer process. (a) Alignment of the WS_2 flake orientation to 0°. (b) Rotation of the WS_2 flake to the desired twist angle, followed by heating prior to engaging the PCL stamp. (c) Contact of the PCL stamp with the flake, cooling to

room temperature, and subsequent pick-up. (d–e) Alignment of the STO substrate to 0° and heating for the drop-down transfer. Figure (f) shows the dry transfer of WS_2 flakes onto oxide substrates using a deterministic dry-transfer setup with rotational control under optical microscopy. This approach allowed us to manually align the flakes with similar angular precision (~1°).

The dry transfer of WS_2 flakes onto (111)-oriented oxide thin films was carried out using a PCL/PDMS stamp[18,19] as shown in Figure S27. The stamp was prepared by spin-coating a thin polycaprolactone (PCL) layer onto a PDMS block. WS_2 flakes exfoliated on Al_2O_3 were aligned under an optical microscope to the desired twist angle and picked up using the heated stamp (Figure S27 (a-c)). The stamp was then brought into contact with the STO(111) substrate, and controlled heating/cooling cycles were applied to release the flakes while minimizing wrinkles or fractures. Finally, residual PCL was removed by immersing the sample in tetrahydrofuran overnight (Figure S27 (d-e)). Figure S27 (f) shows the optical microscopy images of the transferred WS_2 flakes on STO(111) substrates using the deterministic dry transfer method. The images reveal well-defined monolayer WS_2 domains deposited at controlled twist angles. The crystallographic alignment was verified by referencing the WS_2 zigzag edge relative to the $[11\bar{2}]$ direction of the STO(111) substrate. This geometric relation enabled the deterministic control of the twist angle during the transfer process and confirmed the successful fabrication of twisted WS_2/STO(111) heterostructures at the targeted configurations.

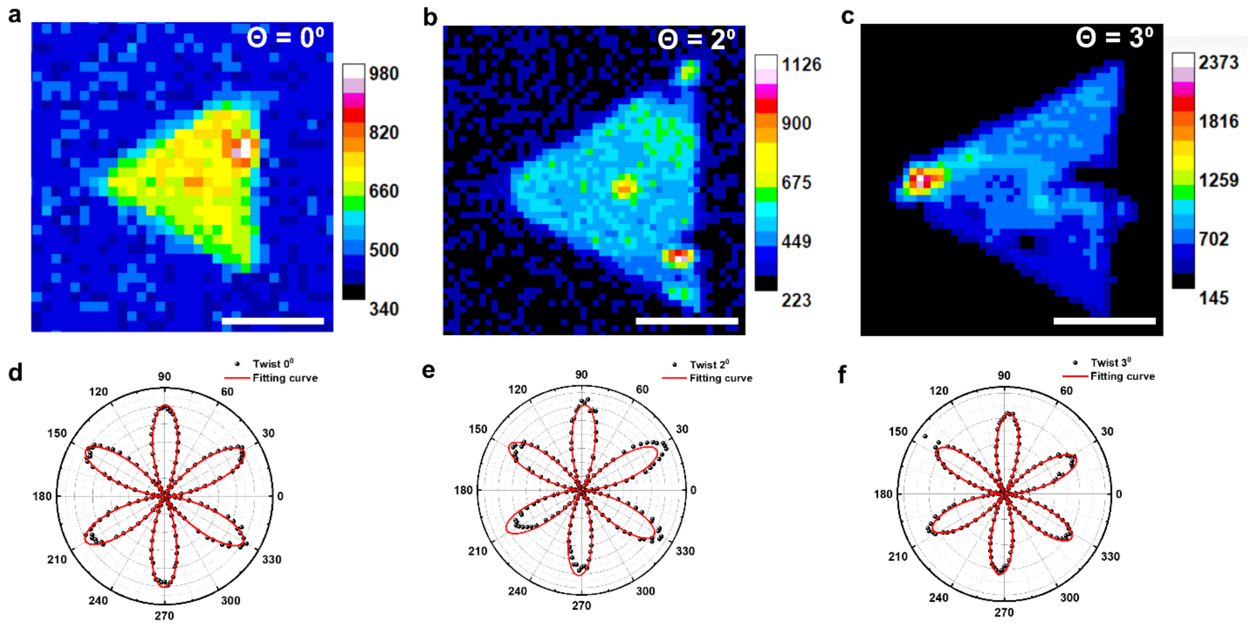

Figure S28 shows the (a-c) SHG mapping with their respective (d-f) polar graphs with angles. The scale bar represents 10µm.

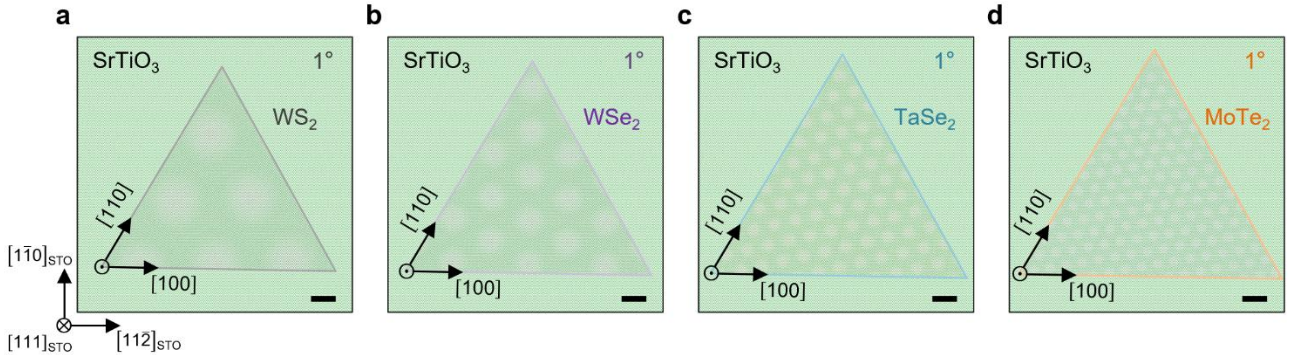

Figure S29 Simulated moiré superlattices of monolayer transition-metal dichalcogenides (MX_2) on $SrTiO_3$ substrates. (a–d) Top-view simulated moiré patterns of WS_2, WSe_2, $TaSe_2$ and $MoTe_2$ monolayers on (111)-oriented $SrTiO_3$ with a 1° rotational mismatch, in which lattice constants of respective TMDs adopted for illustrations are 3.153 Å, 3.29 Å, 3.43 Å, and 3.517 Å, respectively. The periodicity of the moiré lattice decreases with increasing in-plane lattice constant of the overlying MX_2 layer. Sr atoms are represented by green spheres, whereas W, Ta, and Mo atoms are collectively represented by blue spheres. The scale bar represents 5nm.

Supplementary References:

1. Molas, M.R., Nogajewski, K., Potemski, M. *et al.* Raman scattering excitation spectroscopy of monolayer WS_2. *Sci Rep* **7**, 5036 (2017).

2. McCreary, K. M., Hanbicki, A. T., Jernigan, G. G., Culbertson, J. C. & Jonker, B. T. Synthesis of Large-Area WS_2 monolayers with Exceptional Photoluminescence. *Sci Rep* **6**, 19159 (2016).

3.McCreary, K., Hanbicki, A., Singh, S. *et al.* The Effect of Preparation Conditions on Raman and Photoluminescence of Monolayer WS_2. *Sci Rep* **6**, 35154 (2016).

4. Barbone, M. *et al.* Breakdown of the Static Dielectric Screening Approximation of Coulomb Interactions in Atomically Thin Semiconductors, *ACS Nano* **19**, 4269–4278 (2025).

5.Jones, A., Yu, H., Ghimire, N. *et al.* Optical generation of excitonic valley coherence in monolayer WSe_2. *Nature Nanotech* **8**, 634–638 (2013).

6. Weston, A., Zou, Y., Enaldiev, V. *et al.* Atomic reconstruction in twisted bilayers of transition metal dichalcogenides. *Nat. Nanotechnol.* **15**, 592–597 (2020).

7. Rosenberger, Matthew R., *et al.* Twist Angle-Dependent Atomic Reconstruction and Moiré Patterns in Transition Metal Dichalcogenide Heterostructures. *ACS Nano* **14**, 4550-4558 (2020).

8. Bai, Y., Zhou, L., Wang, J. *et al.* Excitons in strain-induced one-dimensional moiré potentials at transition metal dichalcogenide heterojunctions. *Nat. Mater.* **19**, 1068–1073 (2020).

9. Shabani, S., Halbertal, D., Wu, W. *et al.* Deep moiré potentials in twisted transition metal dichalcogenide bilayers. *Nat. Phys.* **17**, 720–725 (2021).

10. Li, H., Li, S., Naik, M.H. *et al.* Imaging moiré flat bands in three-dimensional reconstructed WSe_2/WS_2 superlattices. *Nat. Mater.* **20**, 945–950 (2021).

11. Weston, A., Castanon, E.G., Enaldiev, V. *et al.* Interfacial ferroelectricity in marginally twisted 2D semiconductors. *Nat. Nanotechnol.* **17**, 390–395 (2022).

12. Zhao, S., Li, Z., Huang, X. *et al.* Excitons in mesoscopically reconstructed moiré heterostructures. *Nat. Nanotechnol.* **18**, 572–579 (2023).

13. Weber, M. J. CRC Handbook of Laser Science and Technology. *CRC Press* **4**, (1986).

14. Li, Y. *et al.* Measurement of the optical dielectric function of monolayer transition-metal dichalcogenides: MoS_2, $MoSe_2$, WS_2, and WSe_2. *Phys Rev B* **90**, 205422 (2014).

15. Mennel, L. *et al.* Optical imaging of strain in two-dimensional crystals. *Nat Commun* **9**, 516 (2018).

16. Zhou, H., Chen, Y. & Zhu, H. Deciphering asymmetric charge transfer at transition metal dichalcogenide–graphene interface by helicity-resolved ultrafast spectroscopy. *Sci Adv* **7**, eabg2999 (2021).

17. Ferrante, C. *et al.* Picosecond energy transfer in a transition metal dichalcogenide–graphene heterostructure revealed by transient Raman spectroscopy. *Proceedings of the National Academy of Sciences* **119**, e2119726119 (2022).

18. Kim, Kyounghwan, *et al.* van der Waals heterostructures with high accuracy rotational alignment. *Nano letters* **16**, 1989-1995 (2016).

19. Son, Suhan, *et al.* Strongly adhesive dry transfer technique for van der Waals heterostructure. *2D Materials* **7**, 041005 (2020).
