# Supplementary material for: Crafting moiré superlattices in twisted complex oxide–transition metal dichalcogenide heterostructures
Source: Nat Commun. 2026 Feb 21;17:3025. doi: 10.1038/s41467-026-69773-7 (PMC13036047; doi:10.1038/s41467-026-69773-7)
Supplement: Supplementary file 2 — Description of Additional Supplementary Files [file 41467_2026_69773_MOESM2_ESM.pdf]

Description of Additional Supplementary Files

File name: Supplementary Data 1

Description: This data consists of two folders, ‘special_position’ and ‘gridding’. ‘special_position’ contains AA, AB, and AC stacked full relaxed structure files, while ‘gridding’ contains the full relaxed structure files used to calculate the changes in electronic structure under different in-plane displacement.
